# Directed Assembly of Multi‐Walled Nanotubes and Nanoribbons of Amino Acid Amphiphiles Using a Layer‐by‐Layer Approach

**DOI:** 10.1002/chem.202005331

**Published:** 2021-03-23

**Authors:** Kathrin Siegl, Luba Kolik‐Shmuel, Mingming Zhang, Sylvain Prévost, Kalanit Vishnia, Amram Mor, Marie‐Sousai Appavou, Charl J. Jafta, Dganit Danino, Michael Gradzielski

**Affiliations:** ^1^ Stranski-Laboratorium für Physikalische und Theoretische Chemie Institut für Chemie Technische Universität Berlin Straße des 17. Juni 124 10623 Berlin Germany; ^2^ CryoEM Laboratory of Soft Matter Faculty of Biotechnology and Food Engineering Technion—Israel Institute of Technology Haifa 3200003 Israel; ^3^ Institut Max von Laue-Paul Langevin (ILL) 71 avenue des Martyrs 38042 Grenoble France; ^4^ Faculty of Biotechnology and Food Engineering Technion—Israel Institute of Technology Haifa 3200003 Israel; ^5^ Forschungszentrum Jülich GmbH Jülich Centre for Neutron Science (JCNS) Heinz Maier-Leibnitz Zentrum (MLZ) Lichtenbergerstr. 1 85747 Garching Germany; ^6^ Helmholtz-Zentrum Berlin für Materialien und Energie (HZB) 14109 Berlin Germany; ^7^ CryoEM Laboratory of Soft Matter Faculty of Biotechnology and Food Engineering Technion—Israel Institute of Technology Haifa 3200003 Israel; ^8^ Guangdong Technion—Israel Institute of Technology Guangdong Province Shantou 515063 P. R. China

**Keywords:** amino acid amphiphiles, cryogenic transmission electron microscopy, layer-by-layer assembly, multilayer nanotubes, small-angle neutron scattering

## Abstract

Monodisperse unilamellar nanotubes (NTs) and nanoribbons (NRs) were transformed to multilamellar NRs and NTs in a well‐defined fashion. This was done by using a step‐wise approach in which self‐assembled cationic amino acid amphiphile (AAA) formed the initial NTs or NRs, and added polyanion produced an intermediate coating. Successive addition of cationic AAA formed a covering AAA layer, and by repeating this layer‐by‐layer (LBL) procedure, multi‐walled nanotubes (mwNTs) and nanoribbons were formed. This process was structurally investigated by combining small‐angle neutron scattering (SANS) and cryogenic‐transmission electron microscopy (cryo‐TEM), confirming the multilamellar structure and the precise layer spacing. In this way the controlled formation of multi‐walled suprastructures was demonstrated in a simple and reproducible fashion, which allowed to control the charge on the surface of these 1D aggregates. This pathway to 1D colloidal materials is interesting for applications in life science and creating well‐defined building blocks in nanotechnology.

## Graphical abstract

**Multi‐walled nanotubes and nanoribbons** of amino acid amphiphiles are fabricated by using a layer‐by‐layer technique, where successive rigid amphiphile layers are glued together by oppositely charged polyelectrolyte. The resulting well‐defined 1D structures are attractive as potential biocompatible delivery systems and building blocks in nanotechnology.

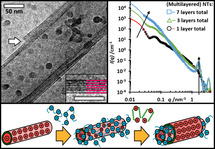

Self‐assembled nanotubes (NTs) and nanoribbons (NRs) have become one of the most interesting topics of recent nanoscience research, as they allow simple access to well‐defined one‐dimensional (1D) colloidal objects. In 1984 Yager and Schoen,^[1]^ Nakashima et al.^[2]^ as well as Yamada et al.^[3]^ independently observed the formation of hollow cylindrical microstructures self‐assembled from different amphiphilic molecules; these structures were termed “tubules”. Frequently encountered building blocks are peptide amphiphiles,^[4]^ where nanotube formation is driven by hydrophobic interactions, chirality and hydrogen‐bonds, which lead to very well‐defined cylindrical structures. They may be formed by the diphenylalanine motif as the essential nanotube forming peptide^[5]^ but in principle, there is quite high flexibility with respect to the selection of the molecular building blocks.^[6]^ The NTs radius depends mainly on the particular molecular architecture of the peptide amphiphile. The radius is typically very uniform and in the range of 5 to 50 nm.^[7]^ Peptide nanotubes are commonly very long and single‐layered. It can be noted that self‐assembled nanotubes can also be formed spontaneously by other systems, such as catanionic amphiphile mixtures containing bile acid,^[8]^ mixtures of sodium dodecyl sulfate (SDS) and cyclodextrine,^[9]^ or from phospholipids by applying electric field.^[10]^ The state of the art of self‐assembly of organic molecules into nanotubes has been reviewed comprehensively by Shimizu.^[11]^ Starting from a heated molecular or micellar (or vesicular) amphiphile solution that is cooled down (often simply to room temperature), nanoribbons and nanotubes form spontaneously by self‐assembly and concurrent chain crystallization due to the intrinsic nature of the building blocks. This simple and spontaneous bottom‐up process then allows access to well‐defined 1D structures that can be used in many applications, including drug delivery^[12]^ and nanotechnology.^[13]^ Of course, of key importance is the ability to control and modify the mesoscopic architecture of these structures as that determines their functional properties. In particular, controlling their wall thickness, or equivalently the number of shells, is scientifically promising as it affects their mechanical properties as well as release properties when structures are loaded with active agents. One simple way of controlling the effective wall thickness is via tuning the chemistry (typically via the length of the hydrophobic part) of the molecules employed, but that is rather limited in scope. For too short chains crystallization does not occur, and for too long chains molecules crystallize always and structures precipitate, without forming 1D structures. An attractive and versatile approach would be to modify those assemblies by going from unilamellar to multilamellar systems. There have been few reports of multilamellar nanotubes, but in these cases the multilamellar structures are formed spontaneously in an uncontrolled manner, thereby leading to structurally rather ill‐defined systems,^[14]^ where the outer layer often shows helical features.^[15]^ Multilayer nanotubes recently have also been employed as drug carriers, where the drug was covalently bound to the tube‐forming catanionic amphiphile mixture.^[16]^

In this work we describe the design of a layer‐by‐layer (LBL) technique in which consecutive addition of the amino acid amphiphile (AAA) to already prepared AAA NTs and NRs correspondingly increases the number of layers, allowing to control the wall thickness while retaining the monodisperse nature in terms of radial extension. Of course, by themselves the AAA would have little tendency to produce a second layer around an already existing nanotube of equal charge, and they remain single‐layered (Figure 1 A). Accordingly, we employed the concept of charge reversal of the surface by depositing a layer of oppositely charged polyelectrolyte. This then facilitates subsequent deposition of another positively charged AAA layer with the polyelectrolyte effectively working as glue between the equally charged AAA layers. By repeating this step‐by‐step approach multi‐walled AAA NTs and NRs can be formed in a well‐controlled manner. Related multilayer systems have been prepared and studied quite extensively for oppositely charged polyelectrolytes, in different shapes (e.g., flat surfaces or microcapsules) and functions.^[17]^ Here we extend this concept and prepare rigid composite 1D materials of AA and polyelectrolyte building blocks.

**Figure 1:**
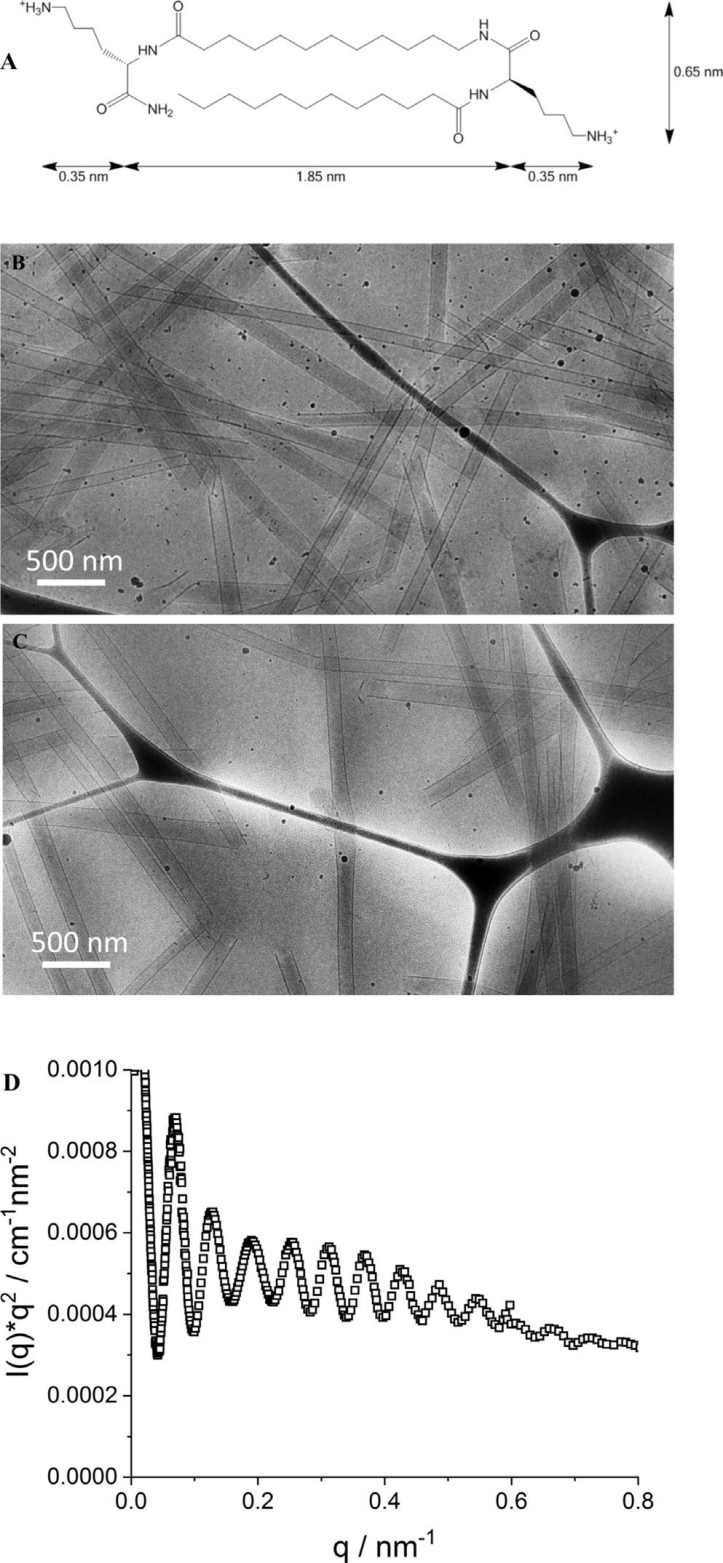
(A) Molecular structure of the nanotube forming AAA C_12_KC_12_K‐NH_2_. For details on intramolecular conformation see Figure S1a. (B) Cryo‐TEM image of 3 mm C_12_KC_12_K‐NH_2_, showing uniform, unilamellar NTs. (C) Identical structures exist also in a control experiment after filtering (which is needed for the LBL process), demonstrating that the filtering does not affect the NT structures. (D) Kratky–Porod plot of SAXS intensity (ID02@ESRF^18^) of a 3 mm C_12_KC_12_K‐NH_2_ solution at pH 8 for nanotubes with a radius of 55.0 nm. Well visible is the high number of oscillations in the data, indicating the low degree of polydispersity (PDI≈0.03, see Supporting Information 4.1 for details) of the radius of the NTs.

For the purpose of spontaneously forming 1D structures we employed the AAA C_12_KC_12_K‐NH_2_ (previously named C_12_β_12_).^[19]^ By bringing a heated solution back to room temperature, long well‐defined NTs are formed spontaneously (Figure 1). Nanotube formation proceeds by chiral self‐assembly from long, thin fibers that immediately self‐assemble after mixing to thin twisted tapes that form within hours by widening of the fiber widths. Tapes further widen to helically coiled ribbons in a matter of days, thereby changing their surface curvature from twisted to helical. This transformation is the result of a delicate interplay between various elastic forces and the chirality of the system.^[20]^ The coiled ribbons then mature over weeks into well‐defined closed nanotubes, with an average inner radius of approximately 50 nm and an average wall thickness of 2.4 nm, which corresponds well to the length of a back‐folded molecule^18^ (see Figures 1 A and S1a).

This thickness was deduced from small‐angle X‐ray and neutron scattering (SAXS, SANS) experiments, and modeling with the suitable form factor for long hollow cylinders as well as by a Kratky–Porod analysis for locally flat structures (see Supporting Information 4.2 and Figure S3 for details; note that in SANS one sees primarily the hydrogenated core of the molecule and less the hydrophilic moieties exposed to the aqueous solution), as previously done for other peptide nanotubes.^[21]^ Both approaches are in good agreement and yield an average wall thickness of unmodified NTs of approximately 2.4 nm. Cryo‐TEM results support these findings and show that over time intermediate fibers and ribbons disappear and eventually very stiff, several microns long NTs are formed, exclusively of a single layer (Figure 1 B, C).

Fully developed NTs remained unchanged for more than two years as confirmed by Cryo‐TEM. The very high monodispersity of the radius (52.5 nm) is best seen in the SAXS experiment presented in Figure 1 D in a Kratky–Porod plot to enhance the visibility of the form factor oscillations done on 2 months old samples. Here, up to 14 form factor oscillations are visible, which indicates the extraordinarily high degree of monodispersity of the radius of the NTs. Modeling the experimental scattering curve with a model of a cylindrical shell (for details see Supporting Information 3.1 and 4.1) indicates a polydispersity index (PDI) of less than 3 % [see Figure S2 and 4.1 for further details including fits with a model of hollow cylinders described by eq. (2)]. It may be noted that this simple shell model does not capture the evolution of the relative amplitude of the oscillations well. This has to be attributed to the fact that in reality the contrast profile is not simply step‐wise, but will have marked features, especially due to the fact that the amphiphilic layer is organized in a crystalline manner. However, relevant for determining the extent of polydispersity is mostly the high number of form factor oscillations and how they are decaying.

These well‐defined unilamellar NTs and NRs then served as templates for depositing subsequent layers of C_12_KC_12_K‐NH_2_ (schematically depicted in Figure 2). In the first step of this preparation procedure the cationic NTs/NRs were treated with sodium poly(methacrylate) (NaPMA) to reverse their charge. After applying a 2‐fold excess of polyanion charges, waiting for 2 days, and careful washing, polyanion modified NRs and NTs were obtained. Next, a new C_12_KC_12_K‐NH_2_ solution was added to these polyelectrolyte‐coated structures, employed again at 2‐fold charge excess. This led within 2 days to the formation of a second C_12_KC_12_K‐NH_2_ layer around the initial structures. Successive steps were done accordingly, alternating between NaPMA and C_12_KC_12_K‐NH_2_, each modification step being followed by a washing cycle. Continuous washing steps proved necessary to minimize the formation of insoluble complexes when adding material for the next coat. A fraction of the structures will precipitate due to net charge compensation with each coating step, thereby decreasing its efficiency. By employing this procedure repeatedly, it was possible to produce NTs and NRs with a well‐defined number of layers, in a fashion not yet done before. In this work, multi‐walled NTs and NRs with a maximum of 7 AAA shells were formed.

**Figure 2:**
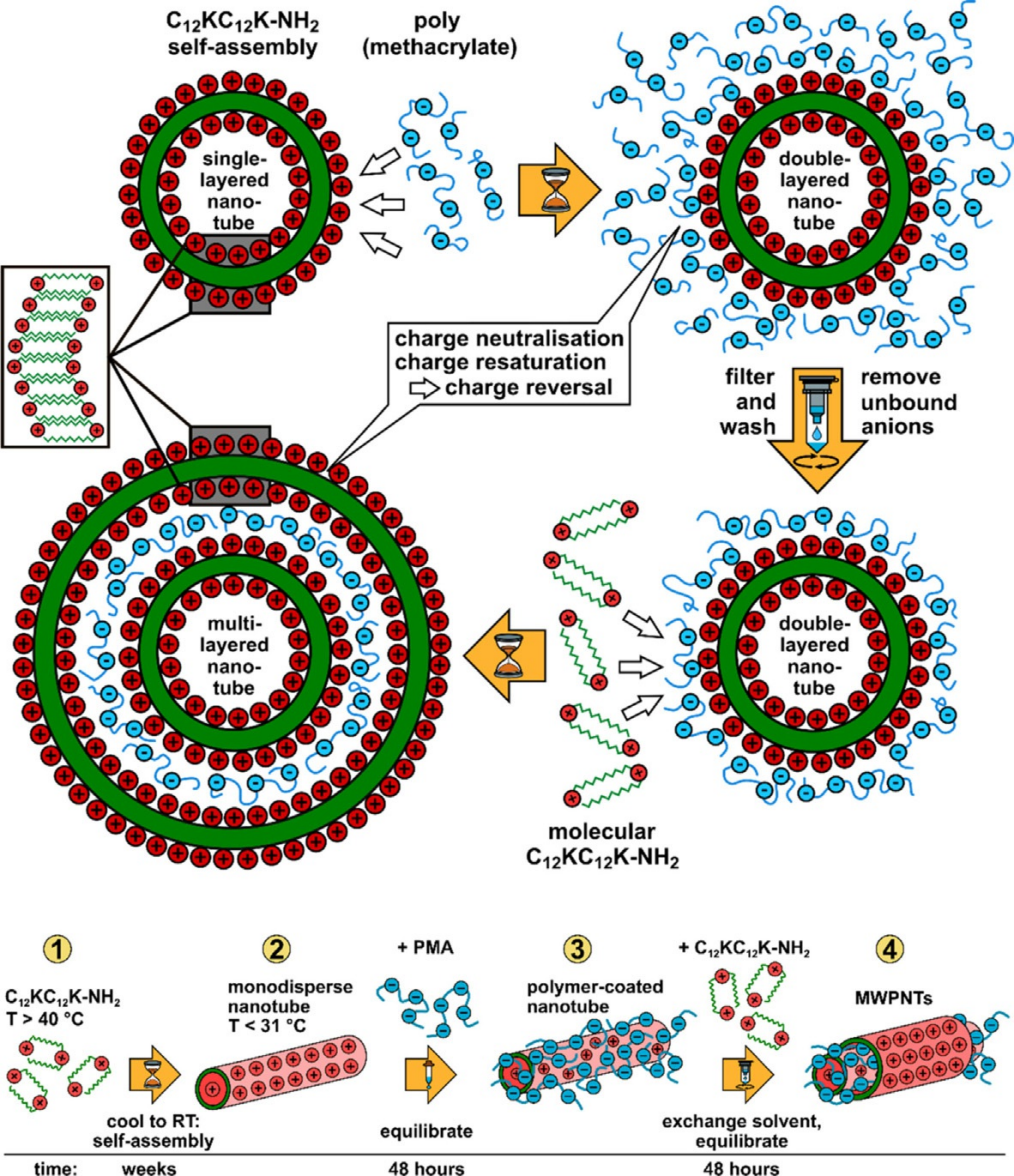
Scheme of the designed route for preparing multi‐layered AAA nanotubes from C_12_KC_12_K‐NH_2_ and NaPMA. Application of one polyelectrolyte and one AAA layer are considered as one modification step. For simplicity only binding to the outer surface is shown, although experiments indicate binding on both sides.

The characterization of these multi‐shell 1D assemblies was done using complementary SANS and cryo‐TEM.^[22]^ Figure 3 a presents SANS curves directly confirming the attachment of subsequent layers as by the increase of intensity in the low q‐range. At the same time the slope of the curves increases substantially, which indicates an effective growth of the thickness of the tube walls. In addition, a correlation peak appears at *q*_m_=1.6–1.7 nm^−1^ (Figure 3 b) which indicates a spacing of the subsequent AAA shells by approximately 3.4–3.8 nm (=2π/*q*_m_). This peak becomes more pronounced with increasing number of deposited shells. It might be noted that in this case Bragg's law (*d*=2π/*q*_m_) is just an approximation as the form factor of the structure walls will also contribute to the scattering pattern, thereby shifting *q*_m_ to a somewhat lower *q* value compared to the pure structure factor peak.

**Figure 3:**
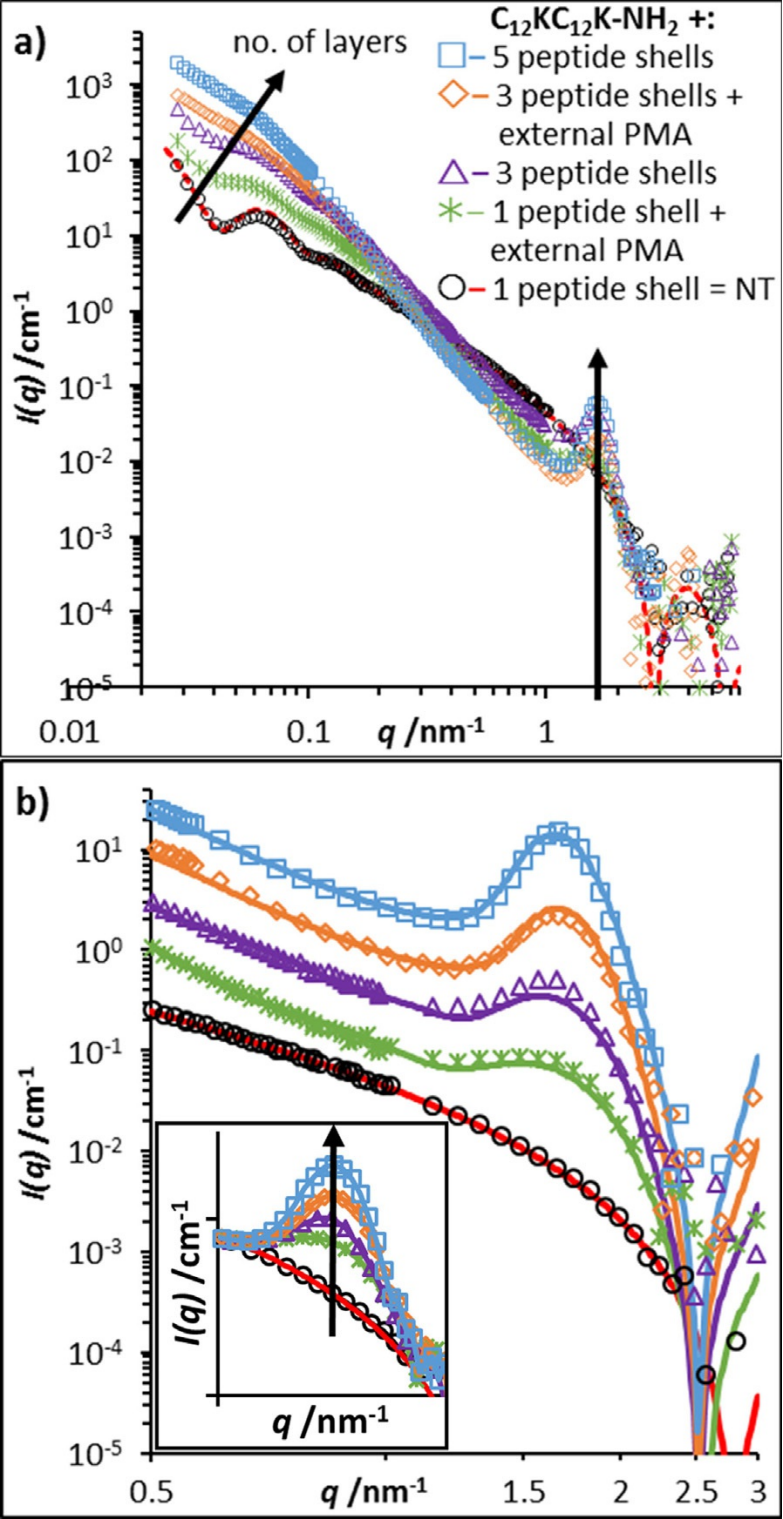
Following the layering process by SANS: (a) Curves for different numbers of deposited coats of NaPMA linking concentric C_12_KC_12_K‐NH_2_ AAA shells, and (b) rescaled data to focus on the appearing Bragg‐peaks with inset showing a direct comparison of the resulting correlation peak heights which were normalized to a common baseline of the unmodified nanotube scattering. Fits (solid lines) were obtained using a model for paracrystalline lamellae on hollow cylinders [see Eqs. (1)–(3)].

Cryo‐TEM corroborates this picture of forming well‐defined layers around the initial unilamellar NRs (Figure 4) and NTs (Figure 4 d). Thus, the cryo‐TEM data reveals multilayered NRs, and confirms that our procedure enables to form a well‐defined number of AAA layers around the initial AAA assemblies.

**Figure 4:**
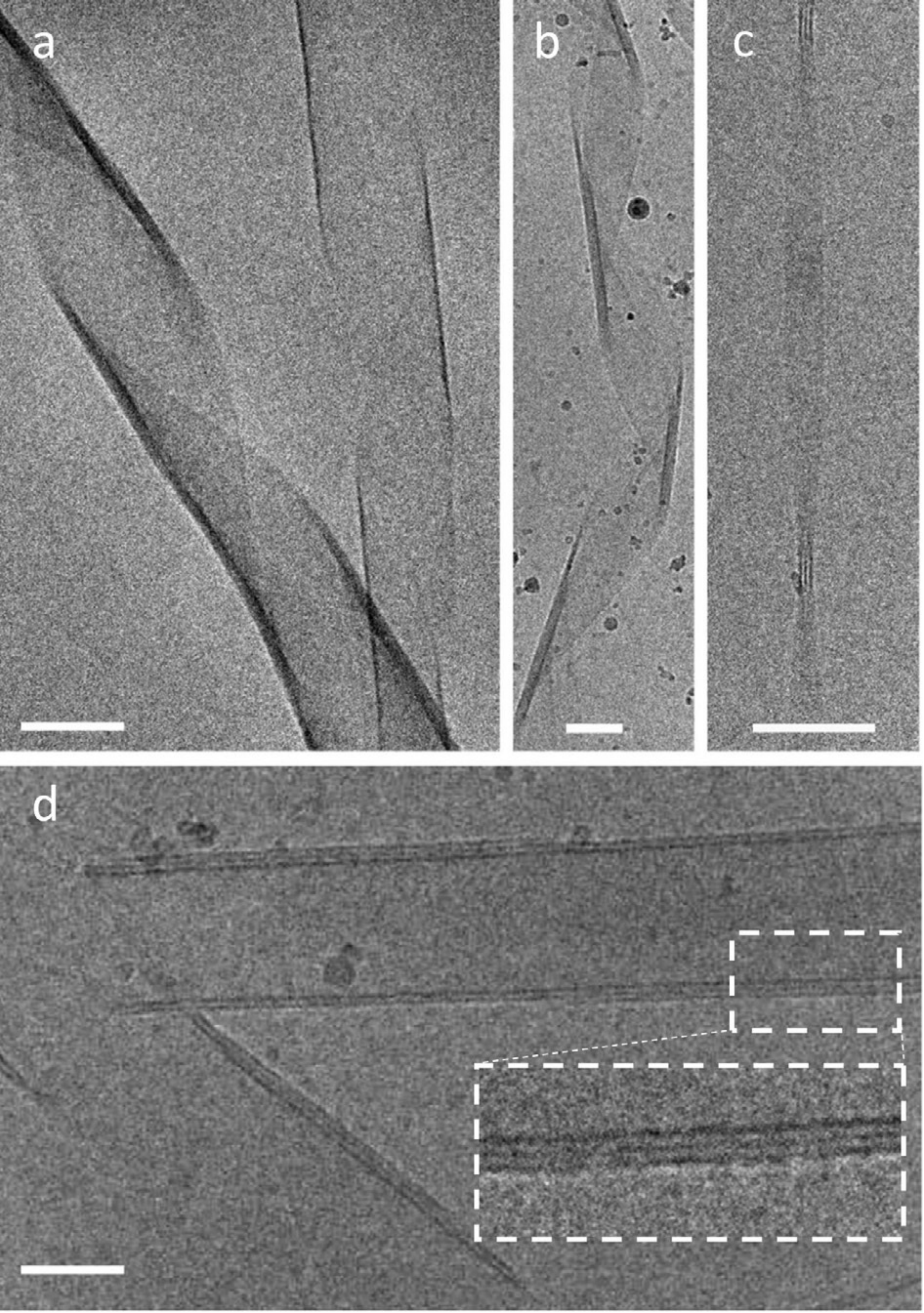
Cryo‐TEM images of multi‐wall NRs composed of alternating AAA (C_12_KC_12_K‐NH_2_) and coat (NaPMA). (a) AAA+coat; (b–d) AAA+coat+AAA; white dashed boxes in (d) are to emphasize the layering in scale and magnified. The initial C_12_KC_12_K‐NH_2_ concentration was always 3 mm. Scale bars are all 50 nm.

The SANS data shown in Figure 3 were quantitatively analyzed with respect to the number of shells, which manifested mainly in the increasing slope of the SANS curves (thicker effective shell) and the correlation peak at *q*≈1.65 nm^−1^ that becomes more pronounced with increasing number of correlated layers due to Bragg scattering. Taking into account the appropriate difference in scattering length densities between the assemblies and the solvent D_2_O (for details see Supporting Information 6.) the overall scattering intensity *I*(*q*) was described by the product of the form factor *P*_cyl_(*q*) of a very long hollow cylinder with a *para*‐crystalline structure factor *S*_PLT_(*q*) that accounts for the correlated layers (see Supporting Information 3.2 for details).^[23]^

The form factor for such a geometry is easily obtained by factorizing it into a cross section form factor *P*_cs_(*q*) for the radial direction and the shape factor *P’*(*q*) for the length direction of the tube, as those two dimensions are virtually uncoupled due to the extreme length of the nanotubes (it might be noted that the samples show anisotropic scattering patterns, see Figure S5)^[24a]^ [Eqs. 1 and 2]:

(1) $$\frac{d\sigma }{d\Omega }\left(q\right)\propto I\left(q\right)≅P_{cyl}\left(q\right)S_{PLT}\left(q\right)=P{}^{'}\left(q\right)P_{cs}\left(q\right)S_{PLT}\left(q\right)$$

(2) $$P_{cs}\left(q\right)=N{\left[\begin{array}{c} {{\left({SLD}_{core}-{SLD}_{shell}\right)\pi R}_{core}}^{2}L\cdot \left(2\frac{J_{1}\left(qR_{core}\right)}{qR_{core}}\right)\\ +{{\left({SLD}_{shell}-{SLD}_{core}\right)\pi (R}_{core}+\Delta R)}^{2}L\cdot \left(2\frac{J_{1}\left[q\left(R_{core}+\Delta R\right)\right]}{q\left(R_{core}+\Delta R\right)}\right) \end{array}\right]}^{2}$$

*R*_core_ is the nanotubes inner (core) radius, Δ*R* its wall thickness, and *L* its length. *J*_1_ is the cylindrical Bessel function of the first kind.

The paracrystalline lamellae theory (PLT) was applied to account for some disorder and lattice defects such as stacking disorder caused by small variations Δ in the average layer separation *d*.[^25a^, ^25b^] The resulting multilamellar arrays are treated as purely one‐dimensional systems along the lattice plane *k*.[^25c^, ^25d^] The theory has long been established with 1D systems and thus is applicable for our case.^[24]^ For further details on these models see Supporting Information 3.2 [Eq. 3]:

(3) $$S_{m,PLT}\left(q\right)=N_{m}+2\sum_{k=1}^{N_{m}-1}\left(N_{m}-k\right)\cos\left(kqd\right)e^{-1/2k^{2}q^{2}\Delta^{2}}$$

The corresponding fits when applying this model are included in Figure 3 and show very good agreement with the experimental SANS data. It might be noted that already a simple multi‐slit model gives generically similar results (see Figure S4), but the paracrystalline model employed here gives more reliable results regarding the average number of layers seen. The deduced parameters are the core radius *R*_core_ and the outer radius *R*_out_, the interlayer spacing *d*, and the number of layers *N*_max_, which are summarized in Table 1 for different numbers of modification steps.

**Table 1:** Parameters obtained from SANS data analysis for a step‐wise modification of self‐assembled 1D structures to obtain ordered mwNTs composed from alternating shells of C_12_KC_12_K‐NH_2_ and NaPMA layers (application of one polyelectrolyte and one AAA layer are considered as one modification step)^[a]^: Maximum number of AAA shells *N*_max_, inner radius *R*_core_, outer radius *R*_out_, layer spacing *d*, molecular weight per unit length determined by SANS (*M*_w,SANS_ *L* ^−1^) and theoretical calculated values (*M*_w,th_ *L* ^−1^) (for details see Supporting Information 5.), and stable coated fraction cf.

Mod. steps	*N*_max_^[a]^	*R*_core_ [nm]	*R*_out_ [nm]	*d* [nm]	*M*_w,SANS_ *L* ^−1^ [g mol^−1^ nm^−1^]	*M*_w,th_ *L* ^−1[b]^ [g mol^−1^ nm^−1^]	cf [wt %]
0	1	53.0	55.4	–	4.1×10^5^	4.3×10^5^	–
1	2.8	49.5	58.8	3.5	1.4×10^6^	1.8×10^6^	80
2	4.9	46.0	62.3	3.6	3.1×10^6^	3.1×10^6^	85

[a] Data were modeled with a form factor for hollow NTs using the paracrystalline lamellae model to obtain the average number of layers comprising one modified nanotube with a layer spacing *d* as well as their average molecular weight per unit length. Layers of C_12_KC_12_K‐NH_2_ have been found to have an average thickness of 2.4 nm, those of NaPMA about 1.15 nm. Scattering length densities: C_12_KC_12_K‐NH_2_=4.24×10^−5^ nm^−2^, NaPMA=1.66×10^−4^ nm^2^. [b] Theoretical molecular weights were calculated based on average layer thicknesses and numbers, assuming a two‐fold charge compensation for NaPMA‐adsorption on the charged surface of C_12_KC_12_K‐NH_2_. Perfect cylindrical geometry of all layers was assumed.

One finds a systematic increase of the average thickness of the NT walls, which goes hand in hand with the increasing number of correlated layers (see Table 1 and Table S1 for more details). This confirms nicely the good control of the multi‐layer structure by our layer‐by‐layer preparation approach. The average outer radius *R*_out_ and inner radius *R*_core_ are roughly changing by the same amount; this indicates that the layering process occurs simultaneously on both the outside and the inside of the NTs, which is confirmed by the direct cryo‐TEM analysis (Figure 4). However, the resulting layers are not always perfectly enveloping the whole structure and can be incomplete, which is also evident from the cryo‐TEM images (box in Figure 4). This results in a slightly varying number of AAA shells along the length axis, seen as step‐like substructures on the mwNTs surface. This also explains why *N*_max_ increases more than the number of steps involved but by slightly less than by a factor of two as would be the case for complete formation of inner and outer AAA layers. The good agreement between the mass per length derived from the SANS intensity data and calculated theoretically (see Supporting Information 5.) is confirming the self‐consistency of our analysis.

Circular dichroism (CD) measurements of various samples were done in order to learn on the internal order of the AAA in the assembled state and the influence of additional polyelectrolyte layers on the structure. Our results demonstrate that the AAA alone (Figure S6) has no classical signal of alpha or beta sheet, but instead a „knee“ structure around 220 nm. The filtration did not change the signal (Figure S6B). Adding a PMA layer also did not change the signal shape, but with further addition of alternate AAA and PMA layers (Figure S6C to F) the „knee“ disappeared and at the same time an increase is seen at low wavelength.

Finally, in order to gain insight into the arrangement of the AAA molecules in the NRs we also performed X‐ray diffraction (XRD) experiments on a sample before and after addition of NaPMA. The spectra are shown in Figure S7 and they indicate the absence of a highly ordered, crystalline packing. The observed main diffraction peak corresponds to a spacing of 0.295 nm and one observes a shift to a smaller spacing by 1.6 % upon addition of the NaPMA. This somewhat denser packing can be explained by the fact that the binding of the oppositely charged PMA on the surface of the nanotubes compensates the charges of the C_12_KC_12_K‐NH_2_ AAA, thereby reducing the electrostatic repulsion of the amino acid head groups. In general, XRD confirms that the crystalline ordering of the C_12_KC_12_K‐NH_2_ AAA is not affected by the presence of the polyelectrolyte.

In summary, we describe a new procedure for the formation of well‐defined multi‐walled AAA nanotubes and nanoribbons by a step‐by‐step, layer‐by‐layer templating approach. For this purpose, first a layer of negatively charged polyelectrolyte (polymethacrylate, NaPMA) was deposited onto the surface of very monodisperse positively charged single‐walled AAA assemblies. Then, these negatively charged NTs or NRs function as a scaffold for the deposition of the next layer of cationic C_12_KC_12_K‐NH_2_ AAA, where the polyanion functions effectively as a glue between the different AAA layers. This process of polyelectrolyte adsorption and AAA deposition can then be repeated several times (done up to a number of 7 shell in total) and the number of repetitions determines the number of AAA layers contained in these mixed composite multi‐walled NRs/NTs. This preparation Scheme is described in Figures 2 and S3. It should be noted that in principle the ability for forming multilayered nanotubes with C_12_KC_12_K‐NH_2_ is quite surprising, as the amphiphile itself forms very monodisperse nanotubes of *R*=55.0 nm and PDI<0.03 (Figures 1 D and S2) but is then able to form tubes with radii of 42.5 and 66 nm for inner or outer shells, respectively, which indicates rather high adaptivity of the peptide shell to allow for other curvatures. Accordingly, an increasing tendency of defects is expected with increasing layer number, as indicated by the cryo‐TEM images.

The layer spacing is very well‐defined at a constant value of approximately 3.6 nm, as evidenced by SANS (Figure 3). Apparently, the process leads to the formation of fully controlled multi‐walled NRs and NTs as depicted in Figure 2. Densely packed layers of AAA are separated by a thin layer (≈1.1 nm) of the polyanionic NaPMA. Interestingly, this shell contains by mass only approximately 15 wt % of hydration water relative to its polyelectrolyte content. It should be noted that depending on the outer layer (AAA or polyanion) the multi‐layer structures are positively or negatively charged. This constitutes an additional degree of flexibility of the formed aggregates, which could be relevant for biomedical applications (especially as one can freely choose the outer polyelectrolyte) but also for other potential applications in material science.

The formed hybrid nanotubes are interesting materials as building blocks in nanoscience due to their being composed of bio‐friendly materials. Their effective thickness (i.e., number of AAA shells) allows to control their robustness, while at the same time retaining a well‐defined porosity via their inner tube opening. Another advantage is that one can have the final mwNT structures with an anionic or cationic cover. Therefore, this work allows for the formation of a colloidal material, as it can be interesting for applications in life science, but also as a well‐controlled building block in nanotechnology. Examples of possible applications would be incorporation of active agents between the amino acid walls and their controlled release (for instance by enzymatic uncoating of the AAA layers), the use of the mwNTs as templates (for instance for metallization), or as multi‐walled channels in nanotechnological devices (where the transport along the cylinder axis could be controlled by the choice of polyelectrolyte).

## Conflict of interest

The authors declare no conflict of interest.

## Supporting information

As a service to our authors and readers, this journal provides supporting information supplied by the authors. Such materials are peer reviewed and may be re‐organized for online delivery, but are not copy‐edited or typeset. Technical support issues arising from supporting information (other than missing files) should be addressed to the authors.

Supplementary
