# Supplementary material for: Directed Assembly of Multi‐Walled Nanotubes and Nanoribbons of Amino Acid Amphiphiles Using a Layer‐by‐Layer Approach
Source: Chemistry. 2021 Mar 23;27(23):6904–10. doi: 10.1002/chem.202005331 (PMC8251557; doi:10.1002/chem.202005331)
Supplement: Supplementary file 1 — Supplementary [file CHEM-27-6904-s001.pdf]

Chemistry–A European Journal

Supporting Information

Directed Assembly of Multi-Walled Nanotubes and Nanoribbons of Amino Acid Amphiphiles Using a Layer-by-Layer Approach

Kathrin Siegl,[a] Luba Kolik-Shmuel,[b] Mingming Zhang,[b] Sylvain Prévost,[c] Kalanit Vishnia,[b] Amram Mor,[d] Marie-Sousai Appavou,[e] Charl J. Jafta,[f] Dganit Danino,*[g, h] and Michael Gradzielski*[a]

1. Materials

Amino acid amphiphile (AAA) $C_{12}KC_{12}K-NH_2$ (Figure S1a) was custom synthesized and purified to ≥98% by Centic Biotec (Heidelberg, Germany) applying the standard procedures for Fmoc solid-phase peptide synthesis.[S1,S2] Poly(methacrylic acid, sodium salt) (NaPMA) was purchased from Sigma-Aldrich as 35 wt% solution in H_2O with an average molecular weight of 9500 g/mol (lot MKBM1345V). NaN_3 was from Sigma-Aldrich at ≥99.5% purity (lot BCBN4225V) and D_2O was purchased from Deutero at 99.9% deuteration (lot B16997).

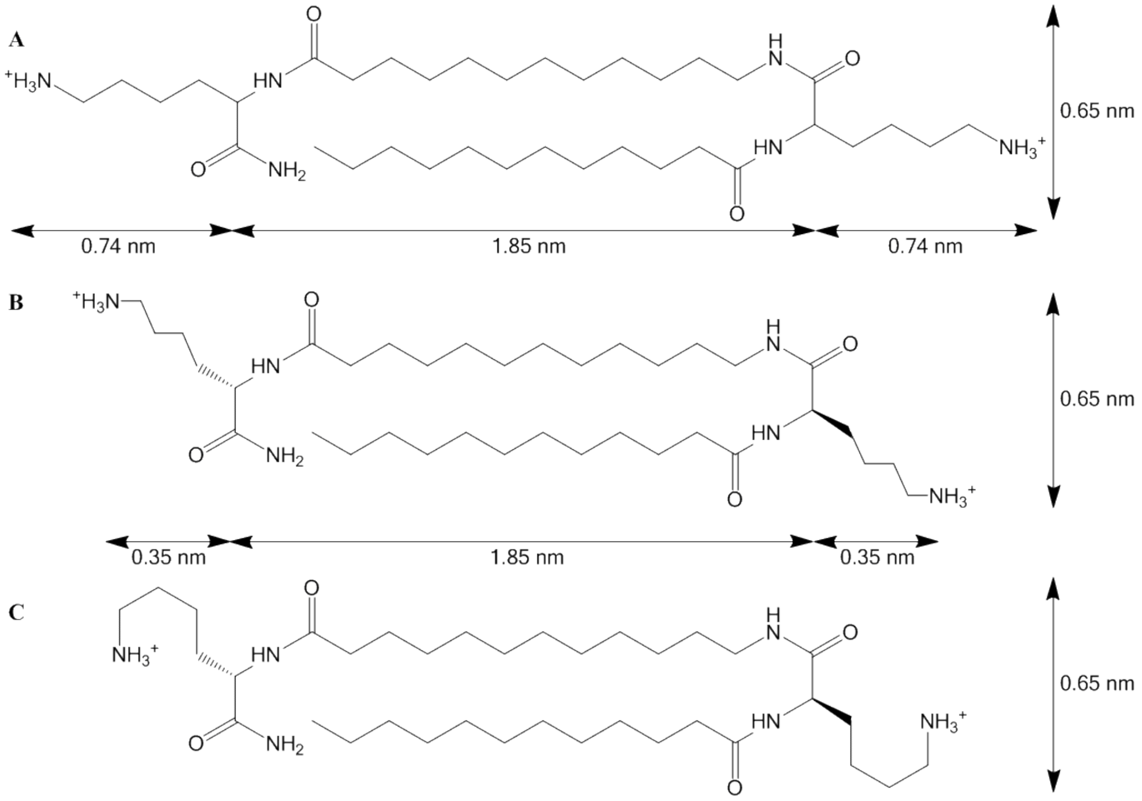

Figure S1a: Molecular structure and proposed probable folds in the L_{β}-gel phase state of the self-assembling AAA $C_{12}KC_{12}K-NH_2$ at room temperature, with indications of the molecular subunits. Structure A has the lysine-residues fully extended due to steric and electrostatic repulsion, whereas B and C aim at surface minimization. In B lysines are angled by about 60°, C shows the lysines folded in on themselves in a gauche-gauche-configuration.

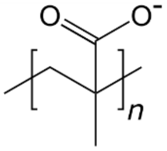

Figure S1b: Molecular structure of poly(methacrylate) used in the preparation of MWPNTs.

2. Experimental Section

2.1 Preparation of Single-Walled Amino Acid Amphiphile NTs and NRs

NRs and NTs were prepared by dissolving lyophilized $C_{12}KC_{12}K\text{-}NH_2$, *i.e.*, $C_{12}\beta_{12}$[S3] in ultrapure water to obtain the desired concentration of 3 mmol/l. 0.05 wt% of NaN_3 was added to all solvents as bactericide. Amphiphile solutions were vortexed for 30 s, sonicated in a bath for 20 min and incubated at 60 °C for 30 min with intermittent vortex to obtain full dissolution of lyophilisate. pH was adjusted to 8 with HCl and NaOH. Samples were heated to 90 °C for 2 h to reverse any possible previous self-assembled structures to a molten micellar state. Subsequently, samples were cooled to 25 °C at a moderate rate (~0.3 °C/min as average cooling rate observed, after placing the samples into room temperature conditions) to allow for a more controlled and ordered self-assembly process, and then incubated at 25 °C for at least one week to allow helical nanoribbons and nanotubes to self-assemble, with longer incubation periods resulting in a higher proportion of mature NTs in solution. Complete self-assembly into fully closed nanotubes can take months.[S3,S4]

For neutron scattering, D_2O was used as solvent instead of water. Here pD was corrected for[S5] and was adjusted as required using DCl/HCl and NaOD.

2.2 Preparation of Multi-Walled Amino Acid Amphiphile NTs and NRs

A defined amount of polyanionic sodium polymethacrylate (NaPMA) was added to solutions containing NTs and NRs aged for a few weeks. The total amount was chosen such that the polyanion charge was twice that of the total number of potential charges of the $C_{12}KC_{12}K\text{-}NH_2$-structures in solution. After NaPMA addition, pH was readjusted to the starting value of pH 8, which was done in order to have comparable and reproducible conditions.

To form another amino acid amphiphile (AAA) layer around the polyanion-modified structures, a concentrated (~ 80 mg/ml) aqueous solution of non-assembled freshly prepared $C_{12}KC_{12}K\text{-}NH_2$ solution was added to previously filtered solutions. To assure its non-assembled state, $C_{12}KC_{12}K\text{-}NH_2$ was dissolved and stored at 60 °C for at least 1 h prior to its addition to the modified samples for which it was cooled close to room temperature. The amount added was again equivalent to twice the number of potential charges of the $C_{12}KC_{12}K\text{-}NH_2$-structures in the original solution. pH was readjusted to the starting value of pH 8. Samples were allowed to equilibrate for a minimum of two days between successive steps.

For samples with multiple coating steps, after each material addition and pH-readjustment, the samples were carefully washed using Amicon© Ultra-2 100k filter units to remove unbound molecules in solution. This was done to minimize the formation of insoluble polyelectrolyte/AAA complexes when adding material for the next coat. After filtration, material for the new coat was added and the solution pH was readjusted accordingly.
Even after careful washing of the samples, a smaller fraction of the MWAAA structures precipitated due to net charge compensation with each coating step, thereby somewhat decreasing process efficiency. The addition of polyanionic coats resulted in a lower observable net stability (*i.e.*, less precipitation) than its cationic AAA counterparts. The whole process is depicted in Figure 2.

2.3 Filtration

1-2 ml per sample was centrifuged at 2000 rpm in a swinging bucket at an angle of 45° rotor until about 50-70% of liquid had passed the filter or for a maximum of 15 minutes. The filtrate was discarded and the sample was filled up with H_2O (D_2O for samples for neutron scattering experiments) to its original starting volume. Filtration was repeated five times or until the liquid volume was fully exchanged at least twice. For sample recovery the filter unit was inverted and centrifuged for 2 min at 1000 rpm in a swinging bucket rotor at 45°. If filtration caused precipitate formation, the resulting precipitate cake was re-dispersed in supernatant by manual shaking or gentle vortex mixing.

2.4 Small angle X-ray Scattering (SAXS)

SAXS data were measured at the beam line ID02[S6] at the European Synchrotron Radiation Facility (ESRF), Grenoble, France. Samples were filled into Kapton capillaries of 2.0 mm diameter. A Rayonix MX-170HS CCD detector was used, operated in low noise mode, with a point-spread-function of ca. 85 µm (2 pixels). Three sample-to-detector distances were selected: 1.5 m (binning 4x4, typical exposure time 7 ms), 6, and 30.7 m (binning 2x2, typical exposure time 300 and 50 ms), at a constant wavelength of 0.0995 nm (relative full width at half mean, FWHM ~ 0.0001) and constant optical settings (to keep a constant beam size at sample position and divergence). Data were corrected from spatial distortion, dark current, flat field and flux ($1.3 \cdot 10^{12}$ photon/s). Transmissions were measured simultaneously with calibrated PIN diodes allowing to calculate the absolute scattering probability per unit solid angle. Absolute scale was verified with the scattering from water at mid and low q, taken to be $1.68 \cdot 10^{-3}$ mm^{-1}. 11 exposures were performed on different positions in the capillaries, and

averaged; errors were estimated from standard deviation. Water in a capillary was subtracted as a background. The absence of radiation damage during the chosen exposure times was verified on selected samples by successive exposures at a constant position.

2.5 Small Angle Neutron Scattering (SANS)

SANS experiments were performed at Heinz Maier-Leibnitz-Zentrum (MLZ) Jülich Centre for Neutron Science (JCNS) instruments KWS-1[S7] and Helmholtz-Zentrum Berlin (HZB) instrument V4 (Berlin, Germany).[S8] Samples were prepared in D_2O and studied at ambient temperature in 2 mm quartz cells 110-QS or 120-QS (Hellma, Germany).

At KWS-1@MLZ three configurations with the wavelengths λ, sample-to-detector distances SD, and collimation lengths C were used: 1) λ=0.5 nm, SD=1.5 m, C=4 m, 2) λ=0.5 nm, SD=8 m, C=8 m, and 3) λ=1 nm, SD=20 m, C=20 m with $\Delta\lambda/\lambda$ =10 % (FWHM).

At V4@HZB four configurations were used: 1) λ=0.45 nm, SD=1.35 m, C=4 m, 2) λ=0.45 nm, SD=6.75 m, C=8 m, 3) λ=0.45 nm, SD=15.75 m, C=16 m, and 4) λ=1 nm, SD=15.75 m, C=20 m with $\Delta\lambda/\lambda$ =10 % (FWHM).

Differential cross sections $d\sigma/d\Omega$ were measured as a function of the scattering vector q with θ being the scattering angle and λ the wavelength of neutrons. Recorded data and detector sensitivities were accounted for by comparison to the scattering of a 1 mm H_2O sample (V4) or a 1,5 mm PMMA (KWS-1) and also used to obtain absolute scale. Scattering data were further corrected for individual sample transmissions and respective backgrounds using the program BerSANS at HZB[S9] and the qtiKWS software at MLZ. Backgrounds subtracted were due to the scattering of the sample cell and resulting scattering intensities still contain contributions of the solvent as well as the incoherent scattering.[S10] As the scattering background varied individually from sample to sample because of their different compositions due to the modification procedures, data quality obtained is better when only using the empty cell signal as generic background scattering and correcting for additional background on a sample-per-sample basis. This was achieved by determining the background level due to incoherent scattering (being strongly dependent on the individual ^{1}H content) of each sample by determination of the slope in a Porod plot ($I(q)q^4 \propto A + I_{bkg}q^4$) and subtracting the resulting backgrounds per sample. Data analysis was done using SASfit.[S11]

2.6 Cryogenic-transmission electron microscopy (cryo-TEM)

The morphology of structures was studied at bulk conditions by preparing vitrified specimens at controlled temperatures and at saturation, and by imaging at low dose conditions.[S12,S13] Typically, 5 µl to 8 µl sample droplets were placed onto Ted Pella perforated TEM grids. Excess liquid was removed by careful blotting, followed by plunging into liquid ethane at its freezing point (-183 °C) to form vitrified samples. The vitrified specimens were then stored in liquid nitrogen (-196 °C) until examination. Analysis at cryogenic temperatures was done using a Tecnai T12-G^2 (FEI, Netherlands) operated at 120 kV, with a Gatan 626 cryo-specimen holder, at temperatures below -173 °C. Images were recorded at low-dose operation on a Gatan MultiScan 791 CCD camera (CM120) or Gatan 2kx2k UltraScan camera (T12-G^2).

2.7 Circular Dichroism (CD)

CD spectra of the different multilamellar nanotube samples were recorded on a JASCO J-1100 spectrophotometer (JASCO, Japan) at ambient temperature, using the supplied Spectra Manager software. Each sample was placed in 0.1 mm quartz cell (Starna Scientific, U.K) and CD spectra were recorded in the wavelength range of 190–300 nm, with 2-3 accumulations for each measurement, data pitch of 1 nm and speed of 25 nm/min. Background CD spectra of water were recorded and subtracted from each spectrum.

2.8 X-Ray Diffraction (XRD)

XRD measurements were done with a PANaytical X´Pert PRO. Measurement time was 22 min and a 2θ scan between 10 and 80° was done in steps of 0.026°. Samples were contained in regular powder sample holders.

3. SANS/SAXS Structure Modeling

The general definition of the differential cross section *dσ/dΩ* for small angle neutron experiments is given by eq. S1. It is a function of the scattering vector *q* and is experimentally measured as the scattering intensity *I(q)*.[S14]

$$\frac{d\sigma}{d\Omega}(q) \propto I(q) = {}^{1}N \cdot V^2 \cdot (\Delta SLD)^2 \cdot P(q) \cdot S(q) + background \qquad \text{(S1)}$$

$$P(q) = \langle |F(q)|^2 \rangle \qquad \text{(S2)}$$

$$q = \frac{4\pi \sin\left(\theta/_2\right)}{\lambda} \qquad \text{(S3)}$$

with θ being the scattering angle and λ the wavelength of the neutrons. ^{1}N represents the number density of scattering particles, V their volume, ΔSLD the contrast in scattering length densities

of dispersed objects to the surrounding solvent. *P(q)* is the form factor, depending on the geometry of the individual scattering aggregates and is defined as the orientational average square of the form factor amplitude *F(q)*, resulting in the isotropic form factor intensity. *S(q)* the structure factor representing interactions between scattering objects. For sufficiently dilute systems, interactions between scattering objects become negligible compared to the shape-dependent contribution *P(q)* and therefore it is assumed that for unmodified nanotubes $S(q)=1$ for $c \rightarrow 0$.

3.1 Hollow Cylinder Form Factor

$C_{12}KC_{12}K\text{-}NH_2$ assembles into very long NTs of micrometers in length, therefore a mathematic simplification for the form factor of a hollow cylinder can be made to minimize computing times and allow for faster analysis.[S15] This is done by factorization of the form factor into a cross section form factor $P_{cs}(q)$ for the shorter dimension, *i.e.* the radial direction of the tube, and a shape factor *P'(q)* for the larger length, direction[S14,S16] (eq. S4). Since length *L* and cross sectional terms (in this case the radius *R*) must be virtually uncoupled in relation, this simplification is only valid for ${}^{L}/_{2R} \gg 1$, specifically ${}^{L}/_{2R} > 5$. In case of $C_{12}KC_{12}K\text{-}NH_2$, nanotube length and diameter differ by two orders of magnitude, therefore its shape can be viewed as an infinitely thin, solid rod along the length axis and can be described by a much simpler term (eq. S5&S6).[S16,S17] The cross sectional form factor itself also simplifies for homogeneous aggregates of centro-symmetric scattering length distributions and local cylindrical geometry with a defined cross sectional radius (eq. S7).

$$\frac{d\sigma}{d\Omega}(q) \propto I(q) \cong P'(q) \cdot P_{cs}(q) \tag{S4}$$

$$P'(q) = \frac{2}{qL}\mathrm{Si}(qL) - \left(\frac{\sin {}^{qL}/_{2}}{{}^{qL}/_{2}}\right)^2 \tag{S5}$$

$$\mathrm{Si}(x) = \int_0^x \frac{\sin(t)}{t}dt \tag{S6}$$

$$P_{cs}(q) = {}^{1}N\left[(SLD_{core} - SLD_{shell})\pi R_{core}{}^{2}L \cdot \left(2\frac{J_1(qR_{core})}{qR_{core}}\right) + (SLD_{shell} - SLD_{core})\pi(R_{core}+\Delta R)^2 L \cdot \left(2\frac{J_1[q(R_{core}+\Delta R)]}{q(R_{core}+\Delta R)}\right)\right]^2 \tag{S7}$$

R_{core} is the inner or core radius of the nanotube, ΔR its wall thickness and J_1 is the cylindrical Bessel function of the first kind.

3.2 Paracrystalline Lamellae Model

When modifying the self-assembled NTs to form MWNTs, *i.e.*, multilamellar structures, the simple form factor approach does not accurately describe the scattering data anymore. Since the general cylindrical shape of present structures does not change, the form factor addressing the geometrical shape will be kept at the simplified factorized hollow cylinder model which describes the data well. The changes in scattering can be addressed by introducing an additional term for the cross sectional scattering contribution. This was done by applying the paracrystalline lamellar model, $S_{m,PLT}$, and a decoupling of the scattering contributions was possible here due to the large size difference between radius and interlamellar spacing of more than a factor of 10. The presence of long-range or quasi long-range order in scattering samples results in the emergence of peaks due to Bragg scattering by the formed multilayers. Whilst in a perfect crystal these would all be of identical width and intensity independent of their order, in real samples some disorder and lattice defects will always be present and need to be represented in the resulting structure factor expansion.[S18,S19] The paracrystalline lamellae theory (PLT) was applied to account for stacking-disorder caused by small variations Δ in the average layer separation d, treating the resulting multilamellar arrays as purely one-dimensional systems along the lattice plane k.[S20,S21]

$$S_{m,PLT}(q) = N_m + 2\sum_{k=1}^{N_m-1}(N_m - k)\cos(kqd)e^{-1/2 k^2 q^2 \Delta^2} \tag{S7}$$

In a paracrystal, the position of a layer and its individual fluctuations is only determined by its nearest-neighbors, therefore losing long-range crystalline order. Bragg-scattering can still be observed due to the presence of quasi long-range order in the stack of N layers with the peaks displaying a typical line shape, rapidly decreasing in peak intensity coupled with progressive broadening for $S_{PLT}(q)$. In a more practical view, this means that a loss of stacking regularity between individual lamellae (increasing stacking disorder Δ) leads to a widening of the peak whilst simultaneously decreasing its amplitude. Incidentally, the peak broadening is more pronounced than the dampening effect. This loss in intensity is usually found as a diffuse background scattering which is also caused due to scattering of a number of uncorrelated layers N_{diff} and is added to the overall scattering intensity via this parameter. Whilst N and d contain

actual structural information on stack size and spacing, Δ and N_{diff} are measures for the degree of order and regularity the stacking process.

The original theory described by Guinier[S20] has been expanded to include this diffuse scattering contributions whilst also incorporating a simplified polydispersity term into the model to eliminate oscillations at low q caused by the theory which have not been observed in experimental data due to inhomogeneities and imperfections in any real system (as well as limited experimental resolutions).[S22] The average of a series of PLT-factors with different numbers of layers in the range $N \pm 2\sigma$ around one layer N_m is weighted by a Gaussian distribution x_m. This can be done within reasonable agreement of the theory and without introducing any new free parameters.

$$S_{PLT}\left(q, N, d, \Delta, N_{diff}\right) = N_{diff} + \sum_{N_m = N-2\sigma}^{N+2\sigma} x_m S_{m,PLT} \tag{S8}$$

$$x_m = \frac{1}{\sigma\sqrt{2\pi}} e^{\left[{(N_m - N)}/{\sigma\sqrt{2}}\right]^2} \tag{S9}$$

$$\sigma = \begin{cases} \sqrt{N} & for\ N \geq 5 \\ {1}/{2}\,(N-1) & for\ 2 \leq N < 5 \end{cases} \tag{S10}$$

The theory of paracrystalline lamellae has long been established with one-dimensional systems and is applicable for the present case of nanotubes.[S23–S26] Taking into account the paracrystalline structure factor than allows for a comprehensive description of the experimentally obtained scattering data of the multi-walled nanotubes.

With this model, all steps in the preparation process, meaning the step of polyelectrolyte addition as well as the step of depositing the next peptide layer, of the multi-walled peptides were analyzed. The obtained fit parameters are summarized in Table S1.

4. Additional results

4.1 Small-angle X-ray Scattering (SAXS)

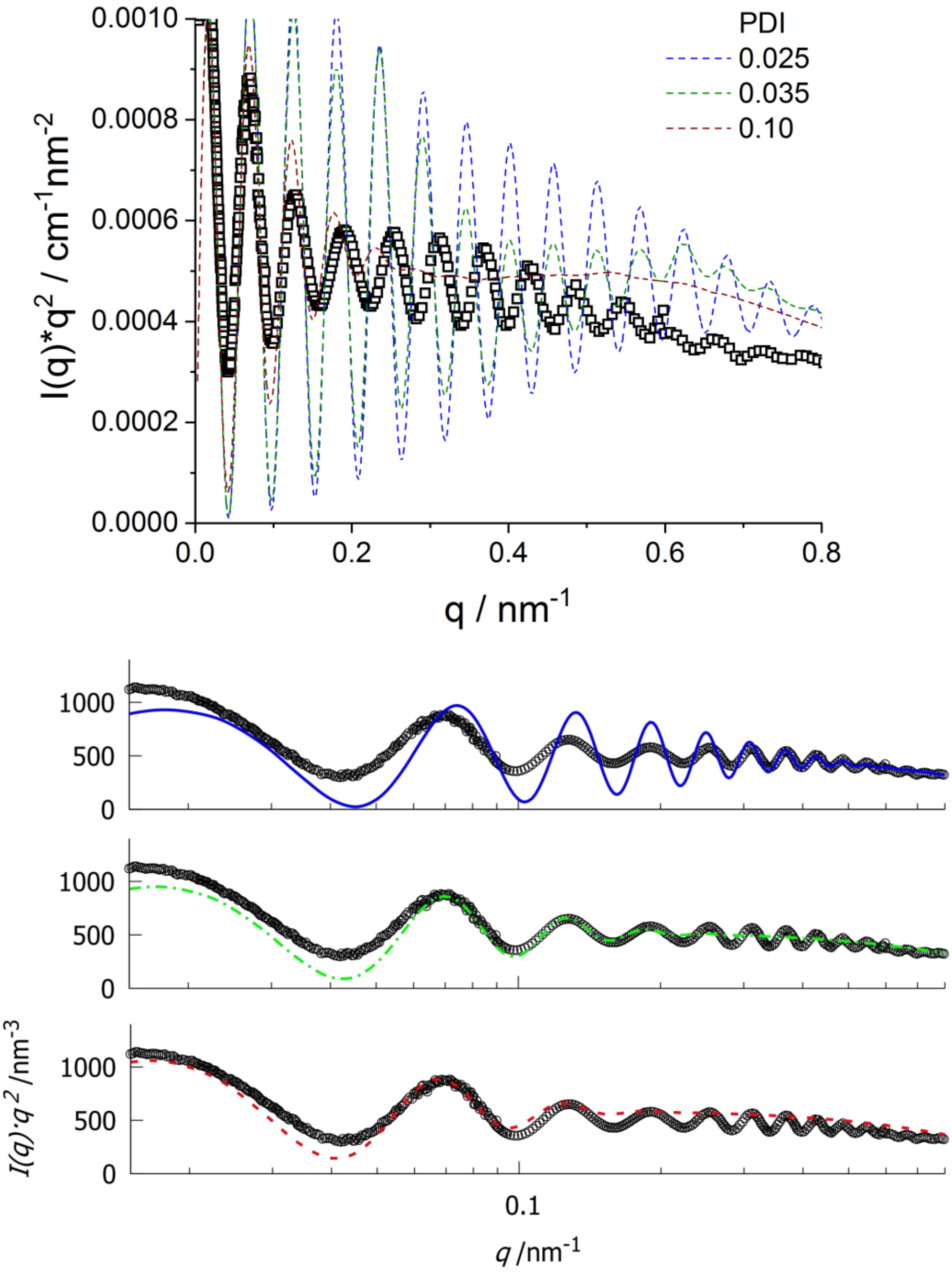

Figure S2. top: Kratky-Porod plot of the X-ray scattering intensity (ID02@ESRF) for self-assembled NTs of $C_{12}KC_{12}K\text{-}NH_2$ at 3 mM solution and pH 8 as a function of the magnitude of the scattering vector q. Corresponding simulation for a hollow cylinder with a mean radius of 55.0 nm, a shell thickness of 3.2 nm and PDI values of 2.5, 3.5 and 10.0%. bottom: fits with the model of hollow cylinders, where the weighting was varied to model different regions of the q-range correctly.

The simulations with a form factor of cylindrical shell shown in Figure S2 demonstrate that one needs a PDI lower than 3.5% in order to account for the high number of oscillations. The overestimation of the first oscillations seen for such low PDI values can be attributed to instrumental smearing and that in reality the scattering length density profile of the amphiphilic shell is not a sharp step function, but will have a structure according to its molecular composition and fluctuations within the structure.

The fits shown on the bottom of Fig. S2 were done using eq. 2 of the main text that describes a hollow cylindrical shell. The focus in the fits was on three different q-ranges, corresponding to the first oscillation [q = 0.05-0.1 nm^{-1}], the 2nd and 3rd oscillations [q = 0.1-0.22 nm^{-1}], and later oscillations [q = 0.34-0.57 nm^{-1}]. Fits were done using a log-normal distribution for the radius and the fit parameters for radius and PDI are, 54.9 nm and 0.150, 53.6 nm and 0.120, and 51.7 nm and 0.047, respectively, for the three different focus ranges. It is clear that this simple model is not able to describe the experimental scattering curves completely. One either can describe the first oscillation well, but then the higher oscillations, which are very well visible, are not described at all by the model, or one has to use a much lower polydispersity to describe well the higher order oscillations, to the detriment of describing the initial oscillations. Apparently this model is too simple to describe well the complete scattering curve, which must have the origin in a more complex scattering length density distribution of the cylinder shell. However, for the polydispersity the oscillations at high q are determining.

4.2 Kratky-Porod Analysis of the Small-angle Neutron Scattering (SANS) Data

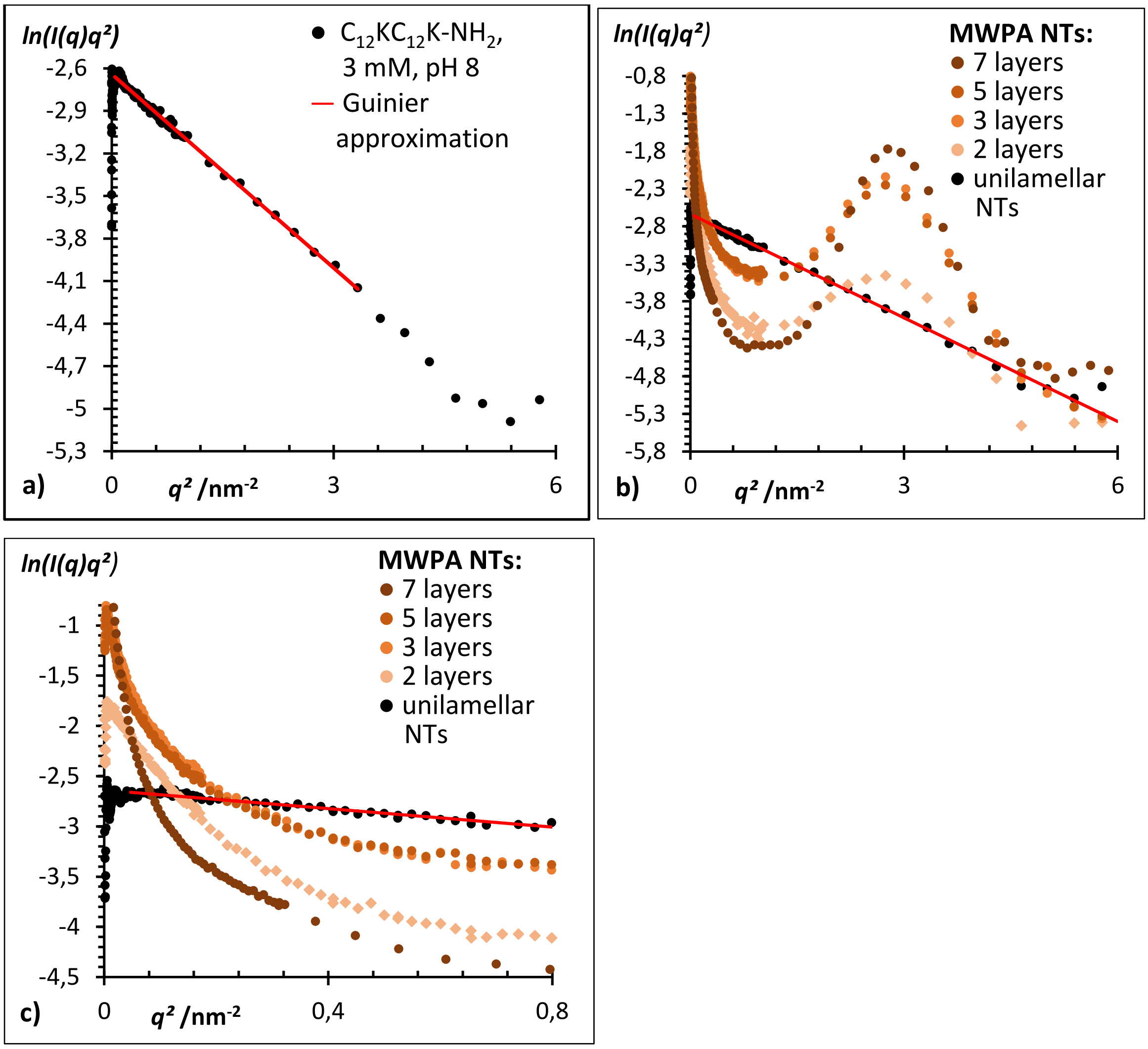

Figure S3: a) Guinier fit for locally flat structures for unmodified $C_{12}KC_{12}K\text{-}NH_2$ NTs. b) A series of modified samples with increasing numbers of layers comprising the multilamellar nanotubes. c) Same data as in b) but focussing on lower q.

In the Kratky-Porod-plot of the unmodified NTs SAXS data, an extended linear region within the data becomes apparent, starting at 0.05 nm^{-2} and going as far as 5 nm^{-2}. Applying the approximation for flat objects (eq. S11)[S20,S27] to this linear regime yielded an average thickness of the nanotube walls of t = 2.4 nm according to

$$I(q) = \frac{I(0)}{q^2} e^{-\left[\frac{q^2 t^2}{12}\right]}. \qquad \text{(S11)}$$

For the modified NTs no linear region is visible and instead differently steep exponential decays appear. This can be interpreted as the presence of a multitude of individual layer thicknesses due to either incomplete layering along the whole length of the NT or a varying

number of layers between individual tubes. A possible linear region at higher q-values is further obscured by appearance of Bragg-peaks in the original data.

4.3 Theoretical Calculation of the Structure Factor with the Multiple Slit Model

In order to explain in an alternative and simple way the observed correlation peaks (e. g. in Fig. 3) we simply calculated the scattering pattern expected for slits as given by eq. S12, which is valid for a number N of identical slits of width b and spacing g.

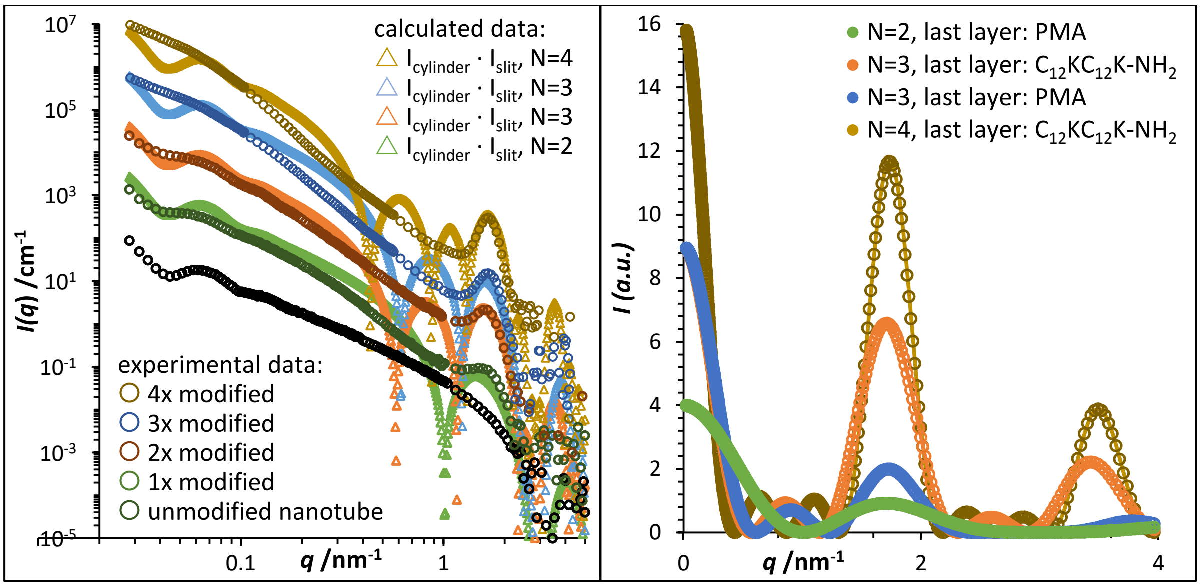

Figure S4: Calculated SANS curves (left) of hollow cylinders when computing intensity profiles (according to eq. S12, shown on the right) for multiple slit diffraction. The slit spacing is g = 3.1 – 3.6 nm with corresponding slit widths of b = 2.3 nm if the outmost layers is NaPMA and b = 1.1 nm for $C_{12}KC_{12}K$-NH_2. The calculated results are compared to experimental data.

$$I(\varphi) = I(\varphi)_{SANS}^{cylinder} \cdot \left(\frac{\sin(\pi\frac{b}{\lambda}\sin(\varphi))}{\pi\frac{b}{\lambda}\sin(\varphi)} \frac{\sin\left(N\pi\frac{g}{\lambda}\sin(\varphi)\right)}{\sin\left(\pi\frac{g}{\lambda}\sin(\varphi)\right)} \right)^2 \text{ with } \varphi = 2\arcsin(\frac{\lambda q}{4\pi}) \text{ (S12)}$$

When calculating the intensity patterns for diffraction on multiple slits or on a diffraction grating and multiplying the results to the intensity profile of an ideal hollow nanotube, the results already are close to those shown experimentally by the multilamellar nanotubes. The appearance of (N-2) side-maxima in the calculations, however, causes strong oscillations that are not visible in the measured SANS-curves. This is because this simple approach (in contrast to the paracrystal lamellae model) does neither account for the inherent polydispersity of the formed structures nor for the non-negligible instrumental smearing of a neutron scattering experiment. In real samples we neither have an ideal diffraction grating nor can we use perfectly monochromatic wavelengths. Additionally, the detection of scattered neutrons is

usually realized by pixel-like detectors that further limit the experimental resolution. However, in general, this simple approach for describing the scattering patterns is rather successful and describes the basic physics behind the observed correlation peaks correctly, which further emphasizes the presence of multiple well-ordered lamellae formed around the initial nanotubes.

4.4 Isotropy of scattering patterns

As the AAA nanotubes are many µm long one may wonder whether this leads to anisotropic scattering patterns, especially in SAXS where the scattering volume is rather small. However, this is basically not the case as depicted in Figure S5 which shows the scattered intensity in 2D representation for a pure AAA NT sample (the azimuthally averaged data of this sample are shown in Figure 1(D)). Figure S5(A) shows the scattering at low q, where one might expect to see best anisotropic effects, while Figure S5(B) shows the larger q-range, where one sees nicely the oscillations of the form factor. However, both plots do not show any obvious indication of anisotropy in the scattering pattern. This demonstrates that even for the relatively small sample volume of 4 nL probed in the SAXS experiment no significant anisotropy is present. Accordingly, for the SANS experiments with their about 10^4 times larger scattering volume any effects due to anisotropy can be completely neglected.

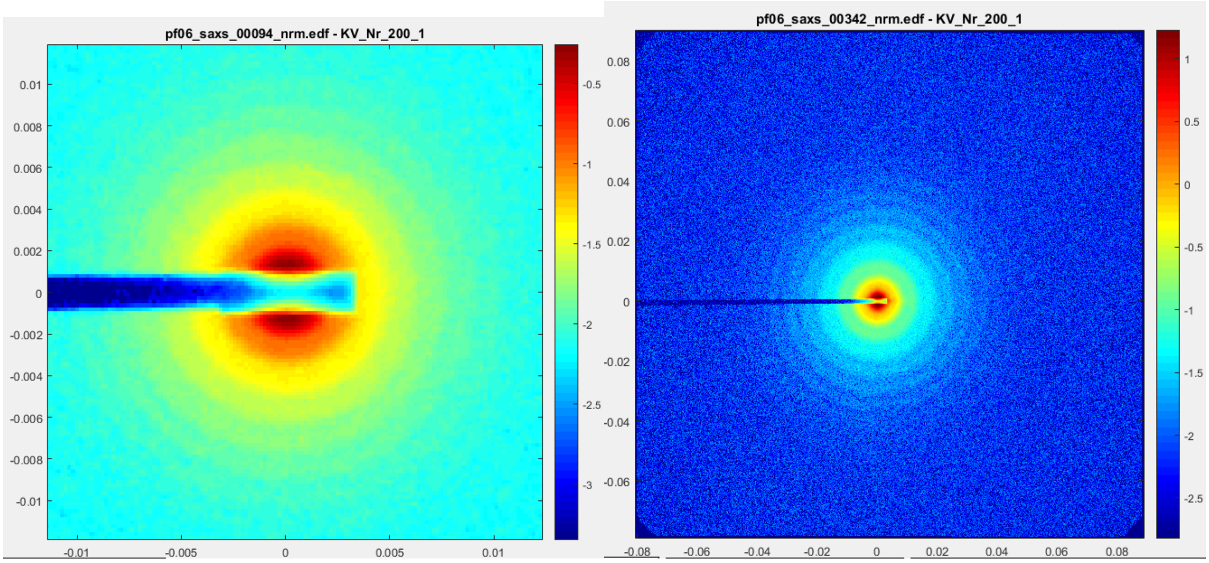

Figure S5: 2D-intensity plots for a 3 mM $C_{12}KC_{12}K$-NH_2 sample in the lower q-region (left) and in the higher q-region (right), which shows nicely the oscillations of the form factor.

4.5 Circular Dichroism (CD)

CD analysis was performed to analyze the order of the chirality-driven self-assembly into nanotubes and nanoribbons, and the effect of incorporating extra layers. The spectra observed are summarized in Figure S6, A to F.

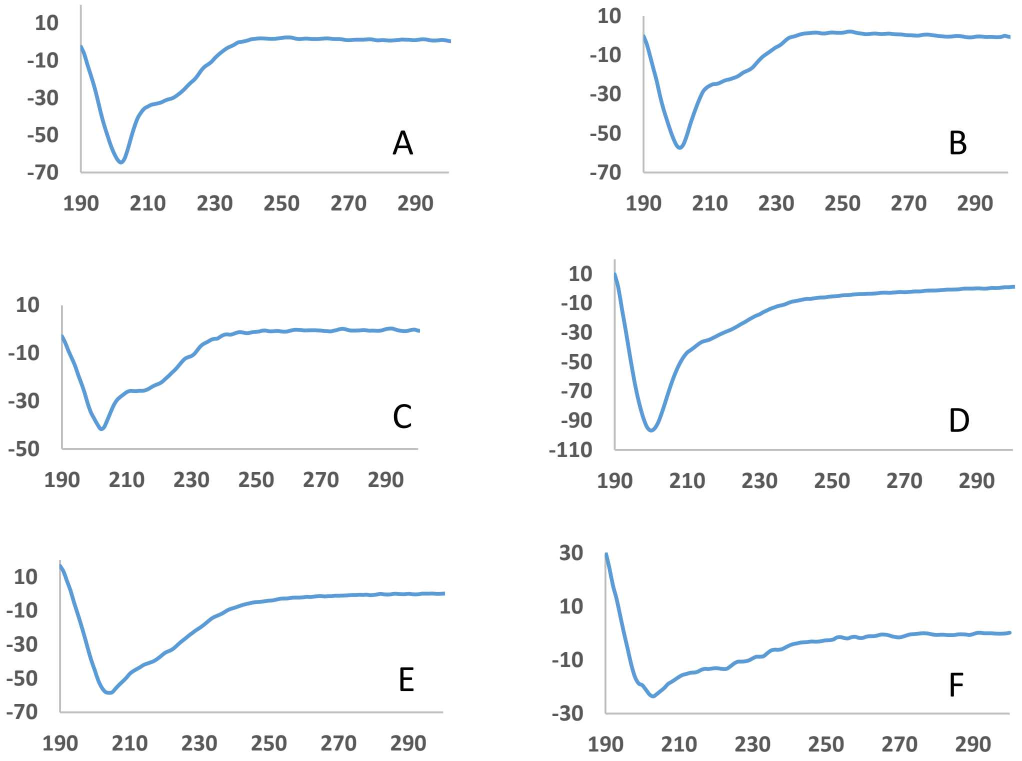

Figure S6: CD spectra of nanotube samples. (A) AAA before filtration; (B) AAA after filtration; (C) AAA+PMA; (D) AAA+PMA+AAA; (E) AAA+PMA+AAA+PMA; (F) AAA+PMA+AAA+PMA+AAA.

4.6 X-ray Diffraction (XRD)

In order to gain additional information regarding the internal structure of the nanotube layers XRD-spectra from a sample with and without added polyelectrolyte (PMA) was measured and the spectra are shown in Figure S6.

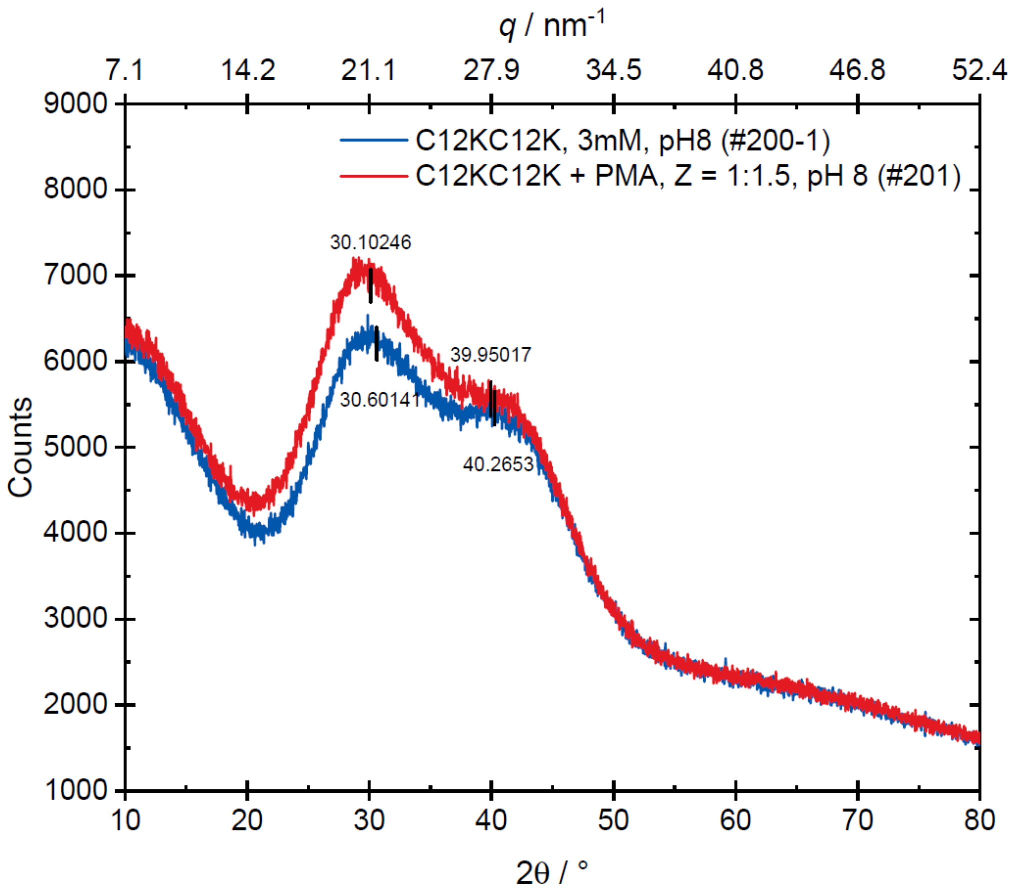

Figure S7: XRD spectra of a sample of 3 mM $C_{12}KC_{12}K$, and of $C_{12}KC_{12}K$ after addition of NaPMA (charge ratio 1:1.5), both samples had a pH of 8.

5. Theoretical Calculation of Mw/length

The calculation of the theoretical molecular weight to compare to the ones derived from small angle scattering was done based on a few simple assumptions. For reasons of simplicity, a perfect cylindrical geometry of each layer within the MWAAA NTs was assumed. To compare data, the average layer thicknesses and numbers from neutron scattering experiments were taken. Layers were modeled to be uniform in thickness and to show an even distribution of mass across their cross-section. It was further assumed that each charge of $C_{12}KC_{12}K-NH_2$ on the nanotube surface was compensated by two PMA monomers (bound in their polymer form). Adsorption was calculated to take place inside and outside of the nanotube.

First, the number of charges Z_{NT} per unit length L on the nanotube surface was calculated as

$$Z_{NT} = \frac{2R_{in,NT}\pi L f_p}{{R_{pep,eff}}^2\pi} z_{NT} e \qquad \text{(S13)}$$

with the inner radius of the nanotube $R_{in,NT}$, the effective radius per AAA molecule $R_{pep,eff}$, the packing fraction f_p, and the charge number $z_{NT} = 2$ of elemental charges e per molecule. Since the shape of the AAA molecule is best approximated by a cylinder, the ideal surface packing will be the molecular arrangement in a hexagonal lattice where each cylinder is surrounded by six other cylinders. The packing fraction of this arrangement is 0.9069.

The effective radius of a molecule accounts for the actual molecular radius as well as the typical spacing between two neighboring molecules in the nanotube crystal. This distance is defined by the typical length of a hydrogen bond[S28] through which the self-assembled structures are stabilized.[S3] Of course, other types of interactions also play a role, namely hydrophobic interactions between the uncharged alkyl-chains in the center of the molecule and electrostatic interactions between the charged head groups.

Since the inner face of the nanotube has a smaller surface than the outside and the molecule building the nanotube packs in a monolayer, the available interior surface limits the maximum number of molecules that can be packed here and thereby sets the total surface charge on both faces.

For the adsorption of PMA, double charge compensation is assumed ($Z_{PMA} = 2Z_{NT}$). As Z and z are linked, their ratio is equal to the number of molecules (or repeat units in case of PMA) contained per layer and unit length. This leads to the theoretical molecular weight for each layer $M_{W,th}$ per unit length:

$$M_{W,th,NT} = \frac{Z_{NT}}{z_{NT}} M_{W,pep} \qquad \text{(S14)}$$

$$M_{W,th,PMA} = \frac{2Z_{NT}}{z_{NT}} M_{W,PMA,mono} \qquad \text{(S15)}$$

with the molecular weight of a AAA molecule $M_{W,pep}$ and repeat unit of PMA, $M_{W,PMA,mono}$.

The total theoretical molecular weight per nanotube und unit length is then simply the sum of all its layers. This outcome of these calculations was then compared to the experimentally determined values and this comparison is given in Table 1.

6. SANS - SLD Profiles

Scattering length densities for all substances used in the production of MWAAA NTs have been calculated based on the atomic composition and densities of the contained molecules. Transitions between adjacent layers have been simplified to sharp boundaries with abrupt changes of corresponding scattering length densities for each layer. This results in a one-step profile for the pre-coating NT, a two-step profile for a polyanion coated NT, a three-step-profile for a MWAAA NT with the NaPMA-layer sandwiched between two AAA layers and so forth. An exemplary profile for a MWAAA nanotube consisting of three AAA layers connected by NaPMA is depicted in Fig. S5.

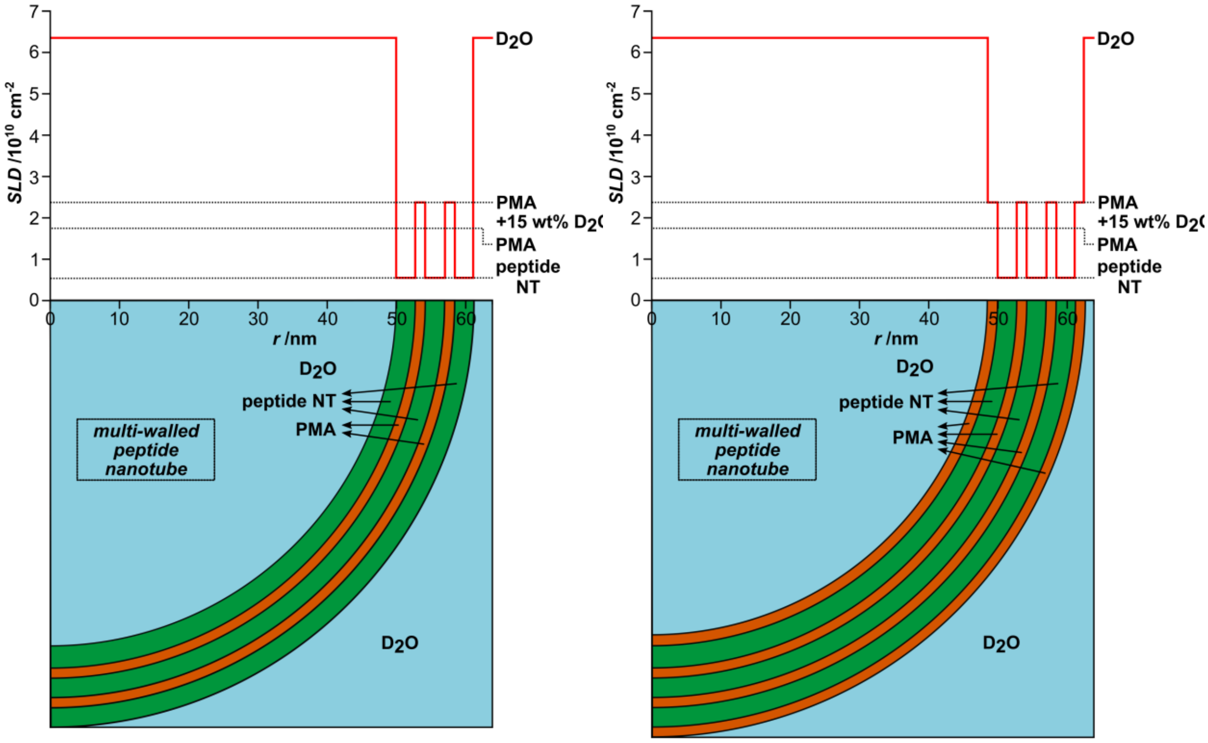

Figure S8: Schematic depiction of a typical step-like scattering length density profile used in SANS modeling for a MWAAA NT consisting of three concentric AAA shells connected by NaPMA-layers with the outside layers being (a) positively charged in case of exterior AAA shells or (b) negatively charged for exterior PMA-layers. PMA-layers contain about 15 wt% of water, thereby increasing the scattering length density of these layers.

6.1 SANS and SAXS - Fit parameters

Table S1: Fit parameters for MWAAA NTs obtained from analyzing SANS (V4@HZV and KWS-1@MLZ) and SAXS (ID02@ESRF) data. For that purpose we used a simplified form factor for hollow cylinders and the structure factor for paracrystalline lamellae. Specific parameters necessary for the hollow cylinder geometry are the radius R_{in} of the initial nanotube as well as its thickness t with a polydispersity σ (as the relative width of the lognormal distribution of radii). Parameters contained in the structure factor are the maximum number of correlated layers N_{max}, the layer spacing d with a stacking disorder parameter Δ and a contribution for the scattering of uncorrelated layers or free polymer N_{diff}. Whilst N_{max} and d contain actual structural information, Δ and N_{diff} are measures for the degree of order and regularity of the coating process.

**Sample composition**	N_{max}	***d*** **/nm**	Δ	N_{diff}	R_{core}* /nm	***t**** /nm	σ*	**SANS/SAXS instrument**
pure NTs	1	--	--	--	53	2.15	≤ 0.1	V4@HZB
pure NTs	1	--	--	--	52.5	2.45	≤ 0.03	ID02@ESRF
NTs + NaPMA	2.4	3.4	0.32	0.25	55	2.4	≤ 0.3	V4@HZB
NTs + NaPMA + AAA	2.8	3.5	0.055	0.2	55	2.4	≤ 0.3	V4@HZB
NTs + NaPMA + AAA + NaPMA	4.7	3.55	0.25	0	55	2.5	≤ 0.5	KWS-1@MLZ
NTs + NaPMA + AAA + NaPMA + AAA	7	3.6	0.3	0	55	2.55	≤ 0.5	KWS-1@MLZ

* For extrapolation to *I(0)* and all theoretical calculations, R_{in} = 53 nm, t = 2.4 nm (obtained via a Kratky-Porod approximation), and σ = 0.1 of the unmodified nanotube have been applied as it can be assumed that the quasi-crystalline nature of the nanotubes is unchanged by the coating procedure. Apparent changes are attributed to incomplete coating and diffuse scattering of unbound and only partially adsorbed polymer chains.

The scattering length densities for alternating layers of AAA and NaPMA were calculated from the atomic composition and density ρ of both compounds with $SLD_{neutrons}(C_{12}KC_{12}K\text{-}NH_2) = 4.24\cdot10^{-5}\ nm^{-2}$, $SLD_{X\text{-}rays}(C_{12}KC_{12}K\text{-}NH_2) = 10.41\cdot10^{-4}\ nm^{-2}$ with $\rho(C_{12}KC_{12}K\text{-}NH_2) = 1.1\ g\cdot cm^{-3}$, and $SLD_{neutrons}(PMA) = 1.66\cdot10^{-4}\ nm^{-2}$, $SLD_{x\text{-}rays}(PMA) = 10.86\cdot10^{-4}\ nm^{-2}$ and $\rho(PMA) = 1.2\ g\cdot cm^{-3}$.

References

(S1) Merrifield, R. B. Solid Phase Peptide Synthesis. I. The Synthesis of a Tetrapeptide. *J. Am. Chem. Soc.* **1963**, *85*, 2149–2154.

(S2) Atherton, E.; Fox, H.; Harkiss, D.; Logan, C. J.; Sheppard, R. C.; Williams, B. J. A Mild Procedure for Solid Phase Peptide Synthesis: Use of Fluorenylmethoxycarbonyl-amino-Acids. *J. Chem. Soc. Chem. Commun.* **1978**, *3*, 537-539.

(S3) L. Ziserman, H. Y. Lee, S. R. Raghavan, A. Mor, D. Danino, *J. Am. Chem. Soc.* **2011**, *133*, 2511.

(S4) L. Ziserman, A. Mor, D. Harries, D. Danino, *Phys. Rev. Lett.* **2011**, *106*, 238105.

(S5) A. Kręzel, W. A. Bal, *J. Inorg. Biochem.* **2004**, *98*, 161.

(S6) P. Van Vaerenbergh, J. Léonardon, M. Sztucki, P. Boesecke, J. Gorini, L. Claustre, F. Sever, J. Morse, T. Narayanan, *AIP Conference Proceedings*; **2016**, 1741, 030034.

(S7) A. V. Feoktystov, H. Frielinghaus, Z. Di, S. Jaksch, V. Pipich, M. S. Appavou, E. Babcock, R. Hanslik, R. Engels, G. Kemmerling, H. Kleines, A. Ioffe, D. Richter, T. Brückel, *J. Appl. Crystallogr.* **2015**, *48*, 61.

(S8) U. Keiderling, A. Wiedenmann, *Phys. B Condens. Matter* **1995**, *213–214* (C), 895.

(S9) U Keiderling, *Appl. Phys. A Mater. Sci. Process.* **2002**, *74*, S1455.

(S10) S. H. Chen, *Annu. Rev. Phys. Chem.* **1986**, *37*, 351.

(S11) I. Breßler, J. Kohlbrecher, A. F. Thünemann, *J. Appl. Crystallogr.* **2015**, *48*, 1587.

(S12) D. Danino, *Curr. Opin. Colloid Interface Sci.* **2012**, *17*, 316.

(S13) D. Danino, H. E. Egelman, *Curr. Opin. Colloid Interface Sci.* **2018**, *34*, 100.

(S14) T. Zemb, P. Lindner, *Neutron, X-Rays and Light. Scattering Methods Applied to Soft Condensed Matter,* 1st ed.; North Holland: Delta Series - Elsevier, 2002, p. 17.

(S15) G. Porod, *Acta Phys. Austriaca* **1948**, *2*, 255.

(S16) T. Neugebauer, *Ann. Phys.* **1943**, *42*, 509.

(S17) A Guinier, G. Fournet, *Small-Angle Scattering of X-Rays*; John Wiley & Sons, Inc.: New York, USA, 1955, p. 49.

(S18) R. Hosemann, S. N. Bagchi, *Direct Analysis of Diffraction by Matter*, 1st ed.; North-Holland Publishing Company: Amsterdam, Netherlands, 1962, p. 302.

(S19) G. Pabst, R. Koschuch, B. Pozo-Navas, M. Rappolt, K. Lohner, P. Laggner, *J. Appl. Crystallogr.* **2003**, *36*, 1378.

(S20) A. Guinier, *X-Ray Diffraction in Crystals, Imperfect Crystals, and Amorphous Bodies*; W. H. Freeman & Company: San Francisco, USA, 1963, p. 309.

(S21) A. E. Blaurock, *Biochim. Biophys. Acta* **1982**, *650*, 167.

(S22) T. Frühwirth, G. Fritz, N. Freiberger, O. Glatter, *J. Appl. Crystallogr.* **2004**, *37*, 703.

(S23) S. Schwartz, J. E. Cain, E. A. Dratz, J. K. Blasie, *Biophys. J.* **1975**, *15*, 1201.

(S24) A. E. Blaurock, J. C. Nelander, *J. Mol. Biol.* **1976**, *103*, 421.

(S25) C. R. Worthington, G. F. Elliott, *Acta Crystallogr. Sect. A* **1989**, *45*, 645.

(S26) M. Bergström, J. S. Pedersen, P. Schurtenberger, S. Egelhaaf, S. *J. Phys. Chem. B* **1999**, *103*, 9888.

(S27) O. Glatter, O. Kratky, *Small Angle X-Ray Scattering*, 1st ed.; Academic Press: London, United Kingdom, 1982, p. 36.

(S28) A. K. Soper, C. J. Benmore, *Phys. Rev. Lett.* **2008**, *101*, 1.
